# Self-care practice and associated factors among patients with diabetes mellitus on follow up at University of Gondar Referral Hospital, Gondar, Northwest Ethiopia

**DOI:** 10.1186/s13104-019-4630-4

**Published:** 2019-09-18

**Authors:** Andualem Yalew Aschalew, Mezgebu Yitayal, Amare Minyihun, Telake Azale Bisetegn

**Affiliations:** 1 Department of Health Systems and Policy, Institute of Public Health, College of Medicine and Health Sciences, University of Gondar, P. O. Box 196, Gondar, Ethiopia; 2 Department of Health Education and Behavioral Sciences, Institute of Public Health, College of Medicine and Health Sciences, University of Gondar, Gondar, Ethiopia

**Keywords:** Diabetes mellitus, Self-care practice, Ethiopia

## Abstract

**Objective:**

Diabetes, a rising global health problem, requires continuous self-care practice. There are limited studies about self-care practice, and most of the studies conducted in Ethiopia focused on some parts of the recommended self-care practices. Therefore, this study aimed to assess diabetes self-care practice and associated factors among diabetic patients attending at University of Gondar Referral Hospital, Gondar, Northwest Ethiopia.

**Results:**

The study revealed that 51.86% (95% CI 46.95–56.72%) of the patients have poor self-care practice. Unable to read and write (AOR = 3.36; 95% CI 1.42–7.90), primary level of education (AOR = 2.62; 95% CI 1.20–5.70), living in rural area (AOR = 3.33; 95% CI 1.61–6.88), having strong social support (AOR = 0.31; 95% CI 0.15–0.62), having diabetes related complication (AOR = 2.20; 95% CI 1.12–4.30), and poor socio-economic status (AOR = 2.16; 95% CI 1.17–3.98) were factors significantly associated with poor self-care practice of patients with diabetes. The study indicates that the prevalence of poor self-care practice was high. Education, residence, socio-economic status, complication and social support were significantly associated with poor self-care practice. Therefore, strategies should be developed to support patients with information, glucometer, and enhance patient’s social support.

## Introduction

Diabetes mellitus (DM) is one of the chronic diseases that affect both developed and developing countries, and it needs lifelong treatment and patient engagement. International Diabetes Federation (IDF) reported that in 2015, Diabetes affected 415 million people worldwide, and it will be 642 million in 2040. An estimated 14.2 million adults aged 20–79 have diabetes in the Sub-Saharan, Africa region. In Ethiopia, it affects 1.3 million people. Its prevalence is around 3.8%, and one of the four priorities of non-communicable diseases [1, 2]. Studies conducted in Northwest Ethiopia showed that the prevalence of DM was 5.1 for urban and 2.1 for rural, and the trend of DM cases in hospitals has increased by 125% over the 10 years observation since 2000 [3, 4].

Ongoing patient self-managements are critical to preventing acute complications and reducing the risk of long-term complications. Successful diabetes self-care requires the following activities: healthy lifestyle, healthy eating, exercise, cessation of smoking, weight management, self blood glucose management, taking medication and foot care. It is the cornerstone to achieving optimal outcomes [5].

Diabetes self-care activities are behaviors undertaken by people with or at risk of diabetes in order to successfully manage the disease on their own. All self-care activities have been found to be positively correlated with good glycemic control, reduction of complications and improvement in quality of life [6].

Various factors: such as sex, age, educational level, social support, presence of complication and co morbidities, and economic status of individuals appeared to influence self-care practices [7–9].

Most of the studies conducted in Ethiopia focused on some parts of the recommended self-care practice which might not give a full picture of the patient’s self care [10, 11]. Moreover, some of the studies did not use standard tools. They also missed some important variables such as wealth status and social support which might be associated with better clinical outcomes and behavioral adaptations. Higher level of social support was associated with increased diabetes self-management and self-care behaviors [12].

From review of the relevant literature, there are limited studies about self care practice and no previous study was conducted in the study area. Therefore, this study aimed to assess self-care practice and its predictors among diabetic patients at University of Gondar Referral Hospital (UoGRH) Diabetic Follow-up Clinic, Ethiopia.

## Main text

### Methods

#### Study design and setting

An institution-based cross-sectional study was conducted to assess self-care practice among patients with diabetes from April to May 2017 at (UoGRH) Diabetic Follow-up Clinic. The hospital is one of the referral hospitals in Ethiopia located in North Gondar Administrative Zone. It has an outpatient department for chronic illness follow-up, and diabetic treatment has been provided 2 days a week.

#### Study population and sampling procedure

The sample size was determined by using single population proportion formula. The proportion of DM self-care practice from the previous study (50.8%) [13] with the assumption of 5% marginal error, 95% confidence interval (CI) and 5% non-response rate. The final sample size was 403. The study population was all adult diabetic patients from Diabetic follow-up Clinic during the study period at UoGRH. All adult diabetic patients on follow-up for at least 6 months and aged ≥ 18 years were included in the study, whereas individuals with gestational diabetes and patients who were unable to communicate were excluded from the study. When the patients came for follow up, their medical record cards were reviewed for the inclusion criteria. Every consecutive patient who met the inclusion criteria were selected until the required sample size was achieved.

#### Data collection procedure

The Summary of Diabetes Self-Care Activities (SDSCA) questionnaire was used to measure the self-care practice. The questionnaire comprises of 10 items with five sub-scale domains. The five sub-scale domains include general diet, specific diet, physical activity, blood glucose testing and foot-care. The SDSCA measures the frequency of performing diabetes self-care activities in the last 7 days. Response choices range from 0 to 7 [14]. The overall mean score was calculated by summation of each item of the scales and divided by the number of items. Therefore, in this study participants who scored equal to or greater than mean score were classified as having good diabetes self-care practice and those who scored below the mean were considered as having poor self-care practice. The questionnaire was developed in Amharic. Factors related to socio-demographics (age, sex, marital status, educational level, occupation, residence, ethnicity, religion and socio-economic status) and clinical data (duration of diabetes, type of diabetes, complications, co-morbidities, body mass index, type of treatment, fasting blood glucose and adherence) were included in the questionnaire. Medication adherence was measured using Morisky Medication Adherence Scale (MMAS‐8) [15] the total score was out of eight, and patients classified as having high adherence = 8, moderate adherence = 6– < 8 and low adherence < 6. Body mass index (BMI) was classified into four categories based on World Health Organization (WHO) classification [16]: underweight < 18.5, normal weight 18.5–24.9, overweight 25–29.9 and obese ≥ 30 [16]. Social Support was measured by the Oslo Social Support Scale (OSSS-3). The total score was calculated by adding up the raw scores for each item. The sum of the raw scores ranges from 3 to 14. A score 3–8 was classified as poor support, a score 9–11 moderate support and a score ≥ 12 strong support [17].

Data on patients’ socio-demographics, self-care practice and some clinical data were collected by a trained interviewer while some clinical data (co-morbidities, complication, diabetes type and fasting blood sugar) were collected from the patients’ medical record card. Complications and co-morbidities were confirmed diagnoses by physicians, and they were written on the patient’s medical card.

#### Data analysis

The collected data were checked for their completeness. Then, data were coded and entered into Epi-Info version 7 Software. Further analysis was done with Stata version 12. Negatively framed question (item 4) was transformed into a positively framed question. For reliability test (Cronbach alpha) was performed to check the reliability of the questionnaire items. Summary statistics were done for the outcome and independent variables. Model assumptions were checked. Binary logistic regression was run to see the crude significant relations of each independent variable with diabetes self-care practice. Variables with p-value ≤ 0.2 in bi-variable logistic regression analysis were entered into multivariable logistic regression. Finally, significant factors were identified based on 95% confidence level adjusted odd ratio (AOR) and p-value ≤ 0.05.

### Results

#### Socio-demographic and economic characteristics of the study participants

A total of 403 (100% response rate) diabetic patients participated. This study indicated that 220 (54.59%) of the participants were male, 135 (33.7%) were unable to read and write, and 282 (69.98%) were from the rural setting (Table 1).

**Table 1:** Socio-demographic and economic characteristics of study participants at UoGRH, Gondar, Ethiopia, 2017 (n = 403)

Variable	Description	Frequency (%)
Sex	Male	220 (54.59)
	Female	183 (45.41)
Age	18–29	60 (14.89)
	30–39	58 (14.39)
	40–49	77 (19.11)
	50–59	106 (26.30)
	> 60	102 (25.31)
Marital status	Single	59 (14.64)
	Married	239 (59.31)
	Windowed	54 (13.40)
	Divorced	51 (12.65)
Occupation	Unemployed	19 (4.72)
	Self-employed	129 (32.01)
	Retired	25 (6.20)
	Government employ	111 (27.54)
	Housewife	102 (25.31)
	Student	17 (4.22)
Educational status	Unable to read and write	135 (33.50)
	Only read and write	57 (14.14)
	Primary education	94 (23.33)
	Secondary education	35 (8.68)
	Above Secondary	82 (20.35)
Residence	Urban	282 (69.98)
	Rural	121 (30.02)
Socio-economic status	Poor	134 (33.25)
	Medium	137 (34.00)
	Rich	132 (32.75)
Social support	Strong social support	147 (36.48)
	Moderate social support	181 (44.91)
	Poor social support	75 (18.61)

#### Clinical characteristics

The study indicated that among the study participants 228 (56.58%) were type 2 diabetes, 115 (28.54%) had co-morbidities, and 85 (21.09%) developed diabetes-related complication (Table 2). The study also revealed that 209 (51.86%) of the study participants had poor diabetic self-care practice.

**Table 2:** Clinical characteristics of study participants at UoGRH, Gondar, Ethiopia, 2017 (n = 403)

Variable	Description	Frequency (%)
Diabetes type	Type 1	175 (43.42)
	Type 2	228 (56.58)
Duration of diagnosis	< 5	155 (38.46)
	5–10	145 (35.98)
	> 10	103 (25.56)
Fasting blood sugar	< 140	133 (33.00)
	≥ 140	270 (67.00)
Type of treatment	Oral medication	141 (34.99)
	Injection	230 (57.07)
	Both	32 (7.94)
Other co-morbidities	Present	115 (28.54)
	Absent	288 (71.46)
DRC	Present	85 (21.09)
	Absent	318 (78.91)
BMI	Under weight	26 (6.45)
	Normal weight	236 (58.56)
	Over weight	108 (26.80)
	Obese	33 (8.19)
Medication adherence	Low adherence	72 (17.87)
	Medium adherence	156 (38.71)
	High adherence	175 (43.42)

*BMI* body mass index, *DRC* diabetes related complication

#### Factors associated with diabetes self-care practice

In the present study, the odds of poor self-care for respondents who were unable to read and write and those with primary level education were 3.36 (AOR = 95% CI 1.42–7.90) and 2.62 (AOR = 95% CI 1.20–5.70) times higher than those with secondary education level and above, respectively. For respondents who come from rural areas, the odds of having poor self-care practice was 3.33 times (AOR = 95% CI 1.61–6.88) higher than those who came from urban areas. The odds of having poor self-care practice was lower by 69% among patients who had strong social support compared to patients who had poor social support. The odds of having poor self-care was 2.20 times higher (AOR = 95% CI 1.12–4.30) among diabetes patients with complication compared to patients without complication. For patients who had poor SES, the odds of having poor self-care practice was 2.16 times higher (AOR = 95% CI 1.17–3.98) compared to patients who had rich SES (Table 3).

**Table 3:** Factors associated with self-care practice among Diabetic patients at UoGRH, Gondar, Ethiopia, 2017 (n = 403)

Variables	Self-care practice		COR (95% CI)	AOR (95% CI)
	Poor n (%)	Good n (%)		
Sex				
Male	107 (51.20)	113 (58.25)	1	1
Female	102 (48.80)	81 (41.75)	1.32 (0.89–1.97)	1.81 (0.92–3.55)
Occupation				
Self-employed	79 (37.80)	50 (25.77)	1	1
Unemployed	11 (5.26)	8 (4.12)	0.87 (0.32–2.31)	1.56 (0.47–5.18)
Retired	10 (4.78)	15 (7.73)	0.42 (0.17–1.01)	2.65 (0.82–8.60)
Government employ	45 (21.53)	66 (34.02)	0.43 (0.25–0.72)	1.33 (0.63–2.84)
Housewife	59 (28.23)	43 (22.16)	0.86 (0.51–1.47)	1.49 (0.62–3.55)
Student	5 (2.39)	12 (6.19)	0.26 (0.08–0.79)	0.59 (0.14–2.38)
Education				
Unable to read and write	93 (44.50)	42 (21.65)	5.35 (2.93–9.74)	3.36 (1.42–7.90)**
Only read and write	34 (16.27)	23 (11.86)	3.57 (1.75–7.27)	2.46 (0.99–6.10)
Primary education	45 (21.53)	49 (25.26)	2.21 (1.18–4.14)	2.62 (1.20–5.70)*
Secondary education	13 (6.22)	22 (11.34)	1.42 (0.61–3.28)	1.15 (0.42–3.07)
Above secondary	24 (11.48)	58 (29.90)	1	1
Residency				
Urban	119 (56.94)	163 (84.02)	1	1
Rural	90 (43.06)	31 (15.98)	3.97 (2.48–6.37)	3.33 (1.61–6.88)**
SES				
Poor	85 (40.67)	49 (25.26)	2.35 (1.43–3.85)	2.16 (1.17–3.98)*
Medium	68 (32.54)	69 (35.57)	1.33 (0.82–2.16)	1.43 (0.78–2.60)
Rich	56 (26.79)	76 (39.18)	1	1
Social support				
Poor support	47 (22.49)	28 (14.13)	1	1
Moderate support	108 (51.67)	73 (37.63)	0.88 (0.50–1.53)	0.95 (0.49–1.84)
Strong support	54 (25.84)	93 (47.94)	0.34 (0.19–0.61)	0.31(0.15–0.62)**
Diabetes type				
Type 1	107 (51.20)	68 (35.05)	1	1
Type 2	102 (48.80)	126 (64.95)	0.51 (0.34–0.76)	0.57 (0.24–1.35)
DRC				
Present	57 (27.27)	28 (14.43)	2.22 (1.34–3.67)	2.20 (1.12–4.30)*
Absent	152 (72.73)	166 (85.57)	1	1
Medication adherence				
Low adherence	48 (22.97)	24 (12.37)	2.26 (1.27–4.02)	1.75 (0.86–3.56)
Medium adherence	79 (37.80)	77 (39.69)	1.16 (0.75–1.79)	1.04 (0.61–1.77)
High adherence	82 (39.23)	93 (47.94)	1	1

*AOR* adjusted odds ratio, *CI* confidence interval, *COR* crude odds ratio, *DRC* diabetes related complication, *SES* socio-economic status

* Variables significant with p-value ≤ 0.05

** Variables significant with p-value ≤ 0.01

### Discussion

This study was conducted to assess the level of self-care practice and predictors among diabetes patients at University of Gondar Referral Hospital.

The study showed that poor self-care practice was 51.86% (95% CI 46.95–56.72%). This finding is in line with the study conducted at Jimma University Teaching Hospital, Southwest Ethiopia 49.1% [13], at Tikur Anbessa Specialized Hospital Addis Ababa, Ethiopia 48.5% [18], the study from Palestine 48% [19] and at Taluk hospital, Bangalore District, India 50.5% [20]. However, the current finding is higher than the findings at Nekemte, Hospital 45% [8], at Addis Ababa Public Hospitals 39.7% [21], at Dilla, University Referral Hospital 23.2% [22], and at Tawam and Al-Ain hospitals, United Arabia Emirates 15.3% [23]. The possible explanations for this difference are differences in source population, for instance, the source population of the Nekemte study include age 15 and above. Moreover, the above studies try to assess self-care practice for only the last 3 days which may be difficult to assess the patient’s self-care behaviour, but the present study asks the last 7 days, which was longer time to capture the number of days the patient’s self-care practice. Finally, this study assessed the number of days individual practice self-care whereas the above studies try to assess self-care practice only by ‘Yes’ or ‘No’ questions.

The current study was lower than the study conducted at three hospitals in Harari region 60.8% [7], 63.6% at Ardabil, University of Medical Science, Iran [24] and at Puducherry, India [25]. This might be due to small sample size as well as the type of tools used to measure the outcome and socioeconomic conditions.

In the present study, for respondents who were unable to read and write and who had primary education level, the odds of poor self-care were 3.36 and 2.62 times higher than that of respondents who had above secondary educational level, respectively. Similar findings were observed in the study conducted at Nekemte and Jimma University Teaching Hospital [8, 13].

Patients who were residents of rural areas had higher odds of having poor self-care practice compared with patients who were residents of urban areas. The possible explanation might be access to diet such as fruits and vegetables, glucometer and information about self-care would be difficult in the rural areas.

Patients who had strong social support had good self-care practice as compared to patients who had poor social support. This was supported by different studies [17, 21]. Possible reasons could be social support from family or friends as a form of emotional, informational or financial can help the patient to cope with problems and give emotional strength.

The odds of having poor self-care was 2.20 times higher among diabetes patients with complication compared to patients without complication. Self-care practice is a good starting point to control blood glucose, and its main outcome is to get good metabolic control. The study showed self-care practices are associated with good metabolic control and reduction of complication [6]. This supports the current finding that patients with diabetes related complication are those who practice self-care poorly.

For respondents who had low SES, the odds of having poor self-care practice was 2.16 times higher as compared to those with high SES. This was supported by the study at Harari region and Nekemte, Ethiopia [7, 8].

### Conclusion

Our findings indicate that the prevalence of poor self-care practice was high. Education, residence, socio-economic status, diabetic related complication and social support were significantly associated with poor self-care practice. Therefore, attention should be given for those patients with complication and poor socio-economic status. Moreover, strategies should be developed to support patients with information, glucometer, and enhance patient’s social support.

## Limitation

The study could not establish a cause and effect relationship due to its cross-sectional nature. The data collection method was self-report rather than direct observation of the patient’s self-care practice which could bias the findings of the study.

## Data Availability

To keep patients’ confidentiality, the raw data would not be shared. But, it is available from the corresponding author on reasonable request and the summary data are available in the main document.

## Abbreviations

ADA: American Diabetic Association
AOR: adjusted odds ratio
BMI: body mass index
CI: confidence interval
COR: crude odds ratio
DM: diabetes mellitus
DRC: diabetes related romplication
Epi info: Statistical Package for Epidemiological Information Analysis
IDF: International Diabetes Federation
SDSCA: Summary of Diabetic Self-Care Activities
SES: socio-economic status
UoGRH: University of Gondar Referral Hospital
WHO: World Health Organization

## References

[1] International Diabetes Federation. IDF Diabetes Atlas, 7th edn. Brussels, Belgium: International Diabetes Federation; 2015.

[2] FMOH. National strategic action plan (NSAP) for prevention & control of non-communicable diseases in Ethiopia. 2014–2016.

[3] Abebe SM, Berhane Y, Worku A, Assefa A (2014). Diabetes mellitus in North West Ethiopia: a community based study. BMC Public Health.

[4] Abebe SM, Berhane Y, Worku A, Alemu S (2013). Increasing trends of diabetes mellitus and body weight: a ten year observation at Gondar university teaching referral hospital, Northwest Ethiopia. PLoS ONE.

[5] American Diabetes Association (2017). 2. Classification and diagnosis of diabetes. Diabetes Care..

[6] Shrivastava SR, Shrivastava PS, Ramasamy J (2013). Role of self-care in management of diabetes mellitus. J Diabetes Metab Disord.

[7] Ayele K, Tesfa B, Abebe L, Tilahun T, Girma E (2012). Self care behavior among patients with diabetes in Harari, Eastern Ethiopia: the health belief model perspective. PLoS ONE.

[8] Amente T, Belachew T, Hailu E, Berhanu N (2014). Self care practice and its predictors among adults with diabetes mellitus on follow up at Nekemte hospital diabetic clinic, West Ethiopia. World J Med Med Sci.

[9] Gautam A, Bhatta DN, Aryal UR (2015). Diabetes related health knowledge, attitude and practice among diabetic patients in Nepal. BMC Endocr Disord.

[10] Berhe KK, Demissie A, Kahsay AB, Gebru HB (2012). Diabetes self care practices and associated factors among type 2 diabetic patients in Tikur Anbessa specialized hospital, Addis Ababa, Ethiopia-a cross sectional study. Int J Pharm Sci Res.

[11] Hailu E, Mariam WH, Belachew T, Birhanu Z (2012). Self-care practice and glycaemic control amongst adults with diabetes at the Jimma University Specialized Hospital in south-west Ethiopia: a cross-sectional study. Afr J Prim Health Care Fam Med.

[12] Strom JL, Egede LE (2012). The impact of social support on outcomes in adult patients with type 2 diabetes: a systematic review. Curr Diabetes Rep.

[13] Kassahun T, Gesesew H, Mwanri L, Eshetie T (2016). Diabetes related knowledge, self-care behaviours and adherence to medications among diabetic patients in Southwest Ethiopia: a cross-sectional survey. BMC Endocr Disord.

[14] Toobert DJ, Hampson SE, Glasgow RE (2000). The summary of diabetes self-care activities measure: results from 7 studies and a revised scale. Diabetes Care.

[15] Morisky DE, Ang A, Krousel-Wood M, Ward HJ (2008). Predictive validity of a medication adherence measure in an outpatient setting. J Clin Hypertens.

[16] Gallagher D, Heymsfield SB, Heo M, Jebb SA, Murgatroyd PR, Sakamoto Y (2000). Healthy percentage body fat ranges: an approach for developing guidelines based on body mass index. Am J Clin Nutr.

[17] Dalgard OS, Dowrick C, Lehtinen V, Vazquez-Barquero JL, Casey P, Wilkinson G, Ayuso-Mateos JL, Page H, Dunn G, Group O (2006). Negative life events, social support and gender difference in depression. Soc Psychiatry Psychiatr Epidemiol..

[18] LEMESSA F: Assessment of self care practices and associated factors among Type 2 diabetic patients at tikur anbessa specialized hospital Addis Ababa, Ethiopia; 2014. http://localhost:80/xmlui/handle/123456789/7038.

[19] Mosleh RSA, Jarrar YB, Zyoud SE, Morisky DE (2017). Factors related to diabetes self-care management behaviors among patients with type II diabetes in Palestine. J Appl Pharm Sci.

[20] Suguna A, Magal A, Stany A, Sulekha T, Prethesh K (2015). Evaluation of self-care practices among diabetic patients in a rural area of Bangalore district, India. Int J Curr Res Aca Rev.

[21] Mamo M, Demissie M (2016). Self care practice and its associated factors among diabetic patients in addisababa public hospitals, cross sectional study. J Diabetes Cholest Metabol.

[22] Addisu Y, Eshete A, Hailu E (2014). Assessment of diabetic patient perception on diabetic disease and self-care practice in Dilla University Referral Hospital. South Ethiopia. J Metabolic Synd..

[23] Al-Maskari F, El-Sadig M, Al-Kaabi JM, Afandi B, Nagelkerke N, Yeatts KB (2013). Knowledge, attitude and practices of diabetic patients in the United Arab Emirates. PLoS ONE.

[24] Nejaddadgar N, Solhi M, Jegarghosheh S, Abolfathi M, Ashtarian H (2017). Self-care and related factors in patients with type 2 diabetes. Asian J Biomed Pharm Sci.

[25] Selvaraj K, Ramaswamy G, Radhakrishnan S, Thekkur P, Chinnakali P, Roy G (2016). Self-care practices among diabetes patients registered in a chronic disease clinic in Puducherry, South India. J Soc Health Diabetes.

